# Human-Pathogenic Kasokero Virus in Field-Collected Ticks

**DOI:** 10.3201/eid2612.202411

**Published:** 2020-12

**Authors:** Amy J. Schuh, Brian R. Amman, Ketan Patel, Tara K. Sealy, Robert Swanepoel, Jonathan S. Towner

**Affiliations:** US Public Health Service Commissioned Corps, Rockville, Maryland, USA (A.J. Schuh);; Centers for Disease Control and Prevention, Atlanta, Georgia, USA (A.J. Schuh, B.R. Amman, K. Patel, T.K. Sealy, J.S. Towner);; University of Pretoria, Onderstepoort, South Africa (R. Swanepoel)

**Keywords:** Viruses, nairoviruses, ticks, Argasidae, Ornithodoros, Chiroptera, Rousettus, bats, ecology, vector-borne infections, arboviruses, Uganda

## Abstract

Kasokero virus (KASV; genus *Orthonairovirus*) was first isolated in 1977 at Uganda Virus Research Institute from serum collected from *Rousettus aegyptiacus* bats captured at Kasokero Cave, Uganda. During virus characterization studies at the institute, 4 laboratory-associated infections resulted in mild to severe disease. Although orthonairoviruses are typically associated with vertebrate and tick hosts, a tick vector of KASV never has been reported. We tested 786 *Ornithodoros* (*Reticulinasus*) *faini* tick pools (3,930 ticks) for KASV. The ticks were collected from a large *R. aegyptiacus* bat roosting site in western Uganda. We detected KASV RNA in 43 tick pools and recovered 2 infectious isolates, 1 of which was derived from host blood–depleted ticks. Our findings suggest that KASV is maintained in an enzootic transmission cycle involving *O*. (*R.*) *faini* ticks and *R. aegyptiacus* bats and has the potential for incidental virus spillover to humans.

The genus *Orthonairovirus* (family *Nairoviridae*) comprises »40 viruses (*1*), including human pathogens such as Crimean-Congo hemorrhagic fever virus. The orthonairoviruses are assigned to 14 species (*1*), most of which have been associated with a single vertebrate subphylum (Vertebrata: bats, birds, rodents, shrews, or ungulates) and tick order (Ixodida: argasids or ixodids) (*2*). The species *Kasokero orthonairovirus* comprises 3 viruses isolated from bats belonging to the suborder Yinpterochiroptera (*3*,*4*) (Pteropodiformes) (*5*) in Africa, namely Yogue virus (YOGV; *Rousettus aegyptiacus*, Senegal), Leopards Hill virus (LPHV; *Hipposideros gigas*, Zambia), and Kasokero virus (KASV; *Rousettus aegyptiacus*, Uganda) (*2*,*6*,*7*).

KASV was first isolated in 1977 by scientists at Uganda Virus Research Institute (UVRI) from 2.7% (2/74) of serum samples collected from *R. aegyptiacus* bats captured at Kasokero Cave in Uganda (*6*). Two months after the KASV bat isolates were introduced to the UVRI laboratory and 3 weeks after the isolates were used in virus characterization assays, a laboratory staff member became ill. Shortly thereafter, 2 additional laboratory staff members became ill. Two of these laboratory staff members had participated in KASV characterization studies that involved virus antigen extraction and serologic testing; the third laboratory staff member had prepared KASV mouse brain suspensions for inoculation and examined virus-infected mice. Sixteen days after symptom onset in the index patient, a UVRI driver who reported no direct contact with the laboratory rooms used to handle the KASV isolates became ill. Manifestations ranged in severity from mild febrile illness to prolonged systemic disease characterized by fever, headache, myalgia, arthralgia, abdominal pain, nausea, diarrhea, chest pain, coughing, and hyperactive reflexes. Intracerebral inoculation of suckling mice with acute phase blood specimens collected from each of the 4 humans yielded a KASV isolate. KASV-specific antibodies were detected in serum from the 4 patients at various times after illness, as well as in 9.5% (10/105) of serum samples collected from other UVRI laboratory staff members and 67.6% (50/74) of the original *R. aegyptiacus* serum. In susceptibility studies, KASV killed suckling and adult mice by intracerebral and intraperitoneal inoculation within 8 days (*6*). In addition, nearly all naive adult mice that nursed KASV-inoculated suckling mice died of KASV infection, indicating horizontal transmission of virus infection.

The home range of *R. aegyptiacus* bats extends throughout sub-Saharan Africa; the bats prefer subterranean environments, such as caves or mines. At multiple locations, *Ornithodoros* (*Reticulinasus*) *faini* ticks (family Argasidae) (*8*) have been observed living within rock crevices and feeding on *R. aegyptiacus* bats (*9*–*13*). Although chiropteran ticks typically exhibit high host-specificity (*9*,*14*), miners, researchers, and other persons entering *R. aegyptiacus* bat roosts have reported being bitten by *O.* (*R*.) *faini* ticks (*13*). Because most orthonairoviruses have been associated with a tick host, *O.* (*R.*) *faini* ticks are likely to be involved in the enzootic transmission and maintenance of KASV and have the potential to be vectors for virus spillover into humans. In 1994 and 1995, KASV was isolated by 1 author (R.S.) from *O.* (*R.*) *faini* ticks collected in Lanner Gorge Cave (22.450°S, 31.150°E) in South Africa, where *R. aegyptiacus* bats roosted. The isolations were made by intracerebral inoculation of suckling mice and identified in cross-neutralization tests in mice using homologous and reference mouse antiserum and the prototype KASV UG Z-52969 isolate obtained from Yale Arbovirus Research Unit (New Haven, CT, USA) and methods described by Shope and Sather (*15*). At the time, KASV was considered a possible bunyavirus, and the isolations remained unpublished. No molecular studies were attempted, and the isolations are no longer available for sequencing. Members of the team that entered Lannar Gorge Cave were bitten by ticks, and in 2 team members, a moderately severe, transient febrile illness developed with headache, malaise, and myalgia a few days later; they refused to seek medical attention or to donate blood samples for virologic examination. 

In this study, we tested 786 tick pools (3,930 total *O.* [*R.*] *faini* ticks) for KASV. We collected the ticks from a large *R. aegyptiacus* bat roosting site in western Uganda in 2013 and 2017.

## Methods

### Tick Collection and Processing

After obtaining approval from the Uganda Wildlife Authority, we collected adult and nymph *O.* (*R.*) *faini* ticks with forceps from rock crevices in Python Cave, Queen Elizabeth National Park, Uganda, over 4 days in April 2013 (*12*) and 1 day in September 2017. A chiropteran population consisting solely of »40,000 *R. aegyptiacus* bats inhabit the cave (*11*). Ticks collected in 2013 were pooled in groups of 5, placed directly into grinding vials (OPS Diagnostics, https://opsdiagnostics.com) containing 250 μL of a 1:1 ratio of MagMax Lysis Binding Solution Concentrate (Thermo Fisher Scientific, https://www.thermofisher.com) to 100% isopropanol (MagMax Lysis Binding Buffer) and then homogenized using the GenoGrinder 2000 (OPS Diagnostics). After we added 550 μL of MagMax Lysis Binding Buffer, we transferred the tick pool lysates to cryovials and stored them under liquid nitrogen (*12*).

Ticks collected in 2017, also pooled in groups of 5, were placed directly into cryovials containing Dulbecco’s Modified Eagle Medium supplemented with 20% heat-inactivated fetal bovine serum (FBS) and antimicrobial drugs and then stored under liquid nitrogen. After thawing the tick pools, we transferred the contents to grinding vials, homogenized them using the GenoGrinder 2000, and then transferred them to a cryovial containing 250 μL of Dulbecco’s Modified Eagle Medium supplemented with 2% heat-inactivated FBS and antimicrobial drugs. We transferred a portion of each tick pool homogenate (100-μL) into a 400-μL aliquot of MagMax Lysis Binding Buffer.

### RNA Extraction and Quantitative Reverse Transcriptase PCR

We extracted RNA (90 μL) from the 2013 (800 μL) and 2017 (500 μL) tick pool lysates using the MagMax Pathogen RNA/DNA Kit on the MagMax Express-96 Deep Well Magnetic Particle Processor (Thermo Fisher Scientific). KASV has an 18.3-kb single-stranded, negative-sense, trisegmented RNA genome comprising large segment that encodes for the viral RNA-dependent RNA polymerase (RdRp), medium segment that encodes for the glycoprotein precursor (GP), and small segment that encodes for the nucleoprotein (N) (*2*). We analyzed RNA by quantitative reverse transcription PCR (qRT-PCR) using the SuperScript III Platinum One-Step qRT-PCR Kit (Thermo Fisher Scientific) with primers and probes (Appendix Table 1) targeting the KASV N gene, tick mitochondrial 16S ribosomal RNA (rRNA) gene (*16*), and eukaryotic 18S rRNA gene (Thermo Fisher Scientific; 2017 tick pools only). Relative KASV RNA copies/tick pool were interpolated from a standard curve generated from a serial dilution of a known concentration of a synthetic KASV RNA oligo.

### KASV Infection Prevalence Calculations

We calculated maximum-likelihood estimates of KASV infection prevalence in individual ticks with exact 95% CIs using an online pooled prevalence calculator (https://epitools.ausvet.com.au). The calculator implemented a frequentist approach and assumed a fixed tick pool size (n = 5) and 100% KASV qRT-PCR sensitivity and specificity (*17*,*18*).

### Virus Isolation and Immunofluorescence Assay

We attempted virus isolation on the four 2017 KASV RNA-positive tick pools. After clarifying the tick pool homogenates (650 μL) by centrifugation, we transferred 200 μL supernatant to a vial containing antimicrobials and incubated it at room temperature for 1 h. Monolayers of Vero E6 cells in 12-well plates were inoculated with 210 μL of antimicrobial-treated supernatant and incubated for 1 h at 37°C under 5% CO_2_. After the addition of 1.3 mL maintenance media, cultures were incubated at 37°C under 5% CO_2_ and monitored daily for cytopathic effect. After 7 d, we transferred 1 mL culture media to a cryovial and replaced with an equal volume of fresh maintenance media. We transferred a portion of the day 7 media (100 μL) into MagMax Lysis Binding Buffer (400 μL) for RNA extraction and qRT-PCR. After 9–10 d, tissue cultures monolayers that were KASV RNA positive at day 7 were scraped to release virus-infected cells. Part of each cellular medium (1 mL) was suspended in 5 mL of borate saline, and 100 μL was placed into MagMax Lysis Binding Buffer (400 μL) for RNA extraction and qRT-PCR. After the cell suspensions were pelleted by centrifugation, the borate saline was decanted, the cells were resuspended in 500 μL borate saline, and 12-well spot slides were spotted with 25 μL of the cellular suspensions. The slides were fixed in acetone before receiving 2 megarads of γ-irradiation.

Six spots on each slide were incubated with 25 μL of a 1:100 dilution of KASV mouse immune ascitic fluid (World Reference Center for Emerging Viruses and Arboviruses, https://www.utmb.edu/gnl/research/wrceva), and the other 6 spots were incubated with normal mouse ascitic fluid for 30 min at 37°C. After the incubation, the spot slides were rinsed 2 times with phosphate buffered saline (PBS), incubated with 24 μL of a 1:40 dilution of goat anti-mouse fluorescein isothiocyanate (MP Biomedicals, https://www.mpbio.com) for 30 min at 37°C, rinsed with PBS, stained with Eriochrome Black T, rinsed with PBS, and then observed under a fluorescence microscope.

### KASV Genome Sequencing

First-strand cDNA was synthesized directly from RNA extracted from 9 of the 2013 KASV RNA-positive tick pools using the qScript XLT cDNA SuperMix Kit (Quantabio, https://www.quantabio.com). KASV amplicons were generated from first-strand cDNA using the Q5 High-Fidelity 2X Master Mix (New England BioLabs, https://www.neb.com) and 6 multiplex pools of KASV-specific tiling primers (Appendix Table 2) that were designed using the Primal Scheme software (http://primal.zibraproject.org) (*19*). We prepared purified KASV amplicons for sequencing using the Accel-NGS 2S DNA Library Kit (Swift Biosciences, https://swiftbiosci.com). Indexed DNA libraries were pooled and then pair-end sequenced using a 500-cycle MiSeq Reagent Kit v2 on the MiSeq System (Illumina, https://www.illumina.com).

After thawing media collected from the two 2017 KASV isolates, we clarified the media by centrifugation and transferred 100 μL supernatant into 400 μL TriPure Isolation Reagent (MilliporeSigma, https://www.emdmillipore.com). We extracted RNA from the KASV isolate lysates using the 5PRIME Phase Lock Gel (Quantabio) system and then purified it using the Monarch Total RNA Miniprep Kit (New England Biolabs). We prepared purified RNA for sequencing using the NEB rRNA Depletion and NEBNext Ultra II RNA Library Kits for Illumina (New England Biolabs). Indexed DNA libraries were pooled and then pair-end sequenced using a 300-cycle MiSeq Reagent Kit v2 on the MiSeq System (Illumina).

### Sequence and Phylogenetic Analyses

KASV sequence data were imported into Geneious 11.1.2 (Biomatters, https://www.geneious.com). After removing KASV-specific primers from the sequences (2013 tick pools), we used BBDuk to trim adaptors and low-quality reads from both sequence ends (minimum quality 30). Long sequence reads were retained (>93% of maximum read length) and normalized to a target coverage level of 40 with a minimum depth of 2. Merged reads were mapped to the concatenated genome sequence of the KASV Z-52963 isolate using the Geneious mapper (minimum mapping quality 30), and consensus sequences were then extracted and parsed according to gene.

We used the MUSCLE algorithm (https://www.ebi.ac.uk/Tools/msa/muscle) to generate N, GP, and RdRp nucleotide and deduced amino acid alignments from the new KASV sequences and existing KASV, YOGV, and LPHV sequences. We constructed maximum-likelihood phylogenies using the PhyML 3.0 algorithm (*20*) in conjunction with the best-fit nucleotide substitution model (*21*) on the ATGC Montpellier Bioinformatics Platform (http://www.atgc-montpellier.fr). We visualized phylogenies using TreeGraph 2 (http://treegraph.bioinfweb.info) (*22*). We estimated the global ratio of the rate of nonsynonymous (d_N_) nucleotide substitutions to the rate of synonymous (d_S_) nucleotide substitutions (d_N_:d_S_) across the KASV nucleotide alignments using the fixed effects likelihood method with the HyPhy version 1.8.2 software (https://www.hyphy.org).

## Results

### Description of the *O.* (*R.*) *faini* Tick Collections

We collected 3,125 *O.* (*R.*) *faini* ticks (625 pools of 5 each) from the rock crevices within *R. aegyptiacus* bat roosting sites in Python Cave, Uganda, in 2013 and 975 *O.* (*R.*) *faini* ticks (195 pools of 5 each) from the same location in 2017. We confirmed the *O.* (*R.*) *faini* species designation by comparative genetic analysis of the 16S rRNA gene of a set of ticks and by morphologic examination (*12*). Screening the *O.* (*R.*) *faini* tick pools for the tick-specific 16S rRNA gene revealed that 4.3% (27/625) of the 2013 pools and 3.6% (7/195) of the 2017 pools were negative, indicating that these samples contained RNA inhibitors and were unsuitable for downstream KASV qRT-PCR analysis.

### Detection of KASV RNA in Ticks

**Figure 1:**
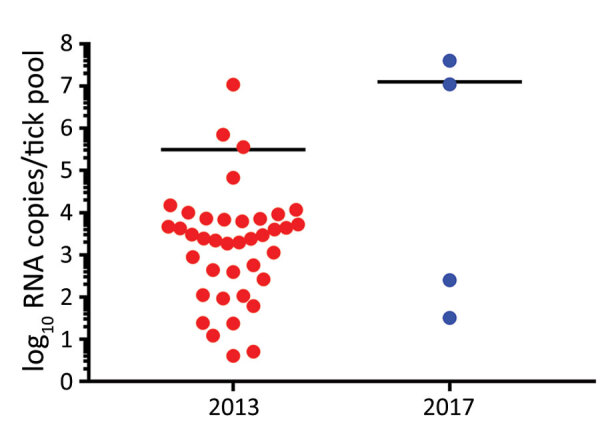
Kasokero virus RNA loads in *Ornithodoros* (*Reticulinasus*) *faini* tick pools from *Rousettus* *aegyptiacus* bats, western Uganda, 2013 and 2017. Black horizontal bars represent mean viral loads.

We detected KASV RNA in 39/598 of the 2013 *O.* (*R.*) *faini* tick pools and 4/188 of the 2017 *O.* (*R.*) *faini* tick pools, resulting in maximum-likelihood estimates of KASV infection prevalence at the individual tick level of 1.34% (95% CI 0.94%–1.83%) and 0.43% (95% CI 0.12%–1.10%), respectively. Based off a standard curve using synthetic KASV RNA, the mean KASV load of the positive 2013 tick pools was 5.5 (range 0.6–7.0) log_10_ RNA copies, and the mean KASV load of the positive 2017 tick pools was 7.1 (range 1.5–7.6) log_10_ RNA copies (Figure 1).

### Isolation of Infectious KASV from Ticks

We isolated infectious KASV from 50% (2/4) of the 2017 KASV RNA-positive tick pools. As expected, the 2 KASV isolates were derived from the tick pools with the highest KASV RNA loads (UGA-Tick-20170048: 7.6 log_10_ RNA copies and UGA-Tick-20170128: 7.0 log_10_RNA copies) (Figure 1). A qRT-PCR targeting the eukaryotic 18S rRNA gene was used to screen the KASV-isolation positive tick pools for *R. aegyptiacus* blood. We found no trace of *R. aegyptiacus* blood in tick pool UGA-Tick-20170048, indicating that the 5 ticks in this pool had not recently taken a blood meal from an actively KASV-infected *R. aegyptiacus* bat. This finding suggests that KASV in this tick pool resulted from active virus replication in >1 tick.

### Circulation of Genetically Diverse KASVs

**Figure 2:**
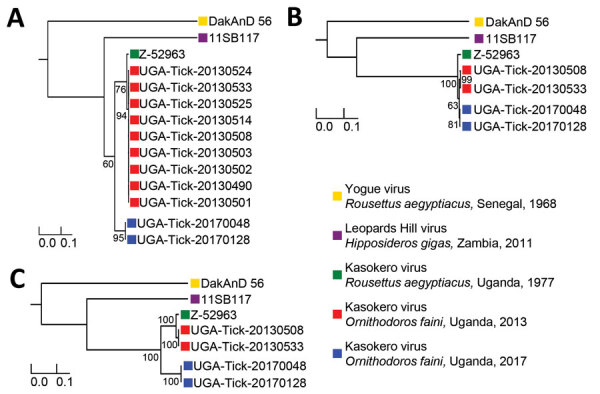
Maximum-likelihood phylogenies constructed from virus sequences belonging to the species *Kasokero orthonairovirus*, including viruses collected from *Ornithodoros* (*Reticulinasus*) *faini* tick pools from *Rousettus* *aegyptiacus* bats, western Uganda, 2013 and 2017. The midpoint rooted phylogenies were generated from complete nucleoprotein (N) (A), glycoprotein precursor (GP) (B), and RNA-dependent RNA-polymerase (RdRp) (C) gene sequences. The N and RdRp gene phylogenies were generated using the general time-reversible nucleotide substitution model with the addition of invariant sites, and the GP gene phylogeny was generated using the general time-reversible nucleotide substitution model with a gamma distribution of rates across sites. Horizontal branch lengths are proportional to the genetic distance between the sequences. Numbers at the end of the branches represent percent bootstrap values based on 1,000 replicates. Only percent bootstrap values >50% are shown. GenBank accession numbers for the new Kasokero virus tick sequences from this study are MT309080–98. Scale bars indicate nucleotide substitutions per site.

We attempted genomic sequencing on 9 of the 2013 KASV RNA-positive tick pools with KASV loads >3.6 log_10_ RNA copies and on the two 2017 KASV tick isolates. We obtained complete sequence coverage for the N (1,545 nt) for 11 of the KASV-positive tick pools, the GP (4,314 nt) for 4 of the KASV-positive tick pools, and the RdRp (11,919 nt) genes for 4 of the KASV-positive tick pools. Maximum-likelihood phylogenies constructed from N, GP, and RdRp gene sequences from the 2013 and 2017 KASV-positive tick pools, as well as prototype KASV (Z-52963, *R. aegyptiacus*, Uganda, 1977), YOGV (DakAnD 56, *R. aegyptiacus*, Senegal, 1968), and LPHV (11SB17, *H. gigas*, Zambia, 2011) isolate sequences, had similar topologies and virus species groupings (Figure 2). Consistent with previous results (*2*,*23*,*24*), YOGV diverged first followed by LPHV, and finally the KASV lineage. The N phylogeny showed 2 distinct KASV lineages defined by >9.2% interlineage nucleotide divergences, and the RdRp phylogeny showed 2 distinct KASV lineages defined by >12.0% interlineage nucleotide divergences; the first lineage included the prototype Z-52963 sequence plus the 2013 tick sequences and the second lineage included the 2017 tick sequences (Appendix Tables 3, 4). Deduced amino acid alignments of the N and RdRp proteins revealed that most KASV lineage-defining nucleotide substitutions were synonymous with interlineage amino acid divergences ranging from 0.6% to 1.0% for the N protein and 2.4% to 2.6% for the RdRp protein. In contrast to the N and RdRp phylogenies, the GP phylogeny shows that the Z-52963 sequence diverged first, followed by the divergence of the 2013 and the 2017 tick sequence groups. Furthermore, the KASV sequences in the GP phylogeny are considerably more similar to one another at the nucleotide level (<2.2% nt divergence and <0.9% aa divergence) and do not form 2 distinct lineages (Appendix Table 5). Consistent with the phylogenetic and KASV gene/protein divergence data, d_N_:d_S_ estimates demonstrated that the N gene was under the strongest purifying selection (0.0110), followed by the RdRp (0.0264) and GP (0.0650) genes.

## Discussion

We detected KASV RNA in 43 *O.* (*R.*) *faini* tick pools collected from a large *R. aegyptiacus* bat colony at Python Cave, Uganda, over a 4-year span. The mean KASV RNA load for the 39 positive 2013 tick pools stored in MagMax Lysis Binding Buffer was lower (5.5 [range 0.6–7.0] log_10_ RNA copies) than the 4 positive 2017 tick pools stored in sterile media supplemented with FBS and antimicrobial drugs (7.1 [range 1.5–7.6] log_10_ RNA copies). Although this difference might have resulted from the choice of sample preservation buffer, it could also be attributed to the type of storage vial (internally threaded for 2017 ticks vs. externally threaded for 2013 ticks), number of RNA freeze-thaw cycles (0 for 2017 ticks vs. 1 for 2013 ticks), month of tick collection in relation to the natural history of KASV infection in *O.* (*R.*) *faini* ticks (September for ticks collected in 2017 vs. April for ticks collected in 2013), or sample size effect (39 KASV-positive tick pools in 2013 vs. 4 KASV-positive tick pools in 2017). We could not attempt virus isolation on the 2013 KASV-positive tick pools because they were placed directly in virucidal buffer; however, we isolated infectious KASV from 2 of the 2017 KASV RNA-positive tick pools. Importantly, molecular evidence of *R. aegyptiacus* blood was not detected in 1 of the KASV isolation-positive tick pools. This finding indicates that the presence of infectious KASV in this tick pool resulted from active virus replication in >1 tick and not from ingestion of a recent blood meal by a tick feeding on a viremic *R. aegyptiacus* bat. Although we did not assess whether KASV can disseminate to the salivary glands of *O.* (*R.*) *faini* ticks and then be successfully transmitted to *R. aegyptiacus* bats, our data coupled with the results of a previous study demonstrating a 2.7% prevalence of active KASV infection and a 67.6% KASV seroprevalence in *R. aegyptiacus* captured at Kasokero Cave, Uganda (*6*), suggest that this virus is maintained in an enzootic transmission cycle involving *R. aegyptiacus* bats and *O.* (*R.*) *faini* ticks. The isolation of KASV from *O.* (*R.*) *faini* ticks collected from a *R. aegyptiacus* bat roost in Lanner Gorge Cave, South Africa, in 1994–1995 (R. Swanepoel, unpub. data) supports this notion and suggests that KASV has a widespread geographic distribution. Tick transmission of KASV also is consistent with our knowledge of the vector status of the orthonairoviruses (*2*). Of the 14 currently recognized orthonairovirus species (*1*), 13 have now been associated with a tick host.

Genetic analysis of the KASV nucleotide alignment showed that the N and RdRp gene sequences were highly divergent, whereas the GP gene sequences were highly conserved. However, the low d_N_:d_S_ estimates, together with the high level of conservation between the deduced amino acid sequences for the N, GP, and RdRp proteins, suggest that strong purifying selection purged deleterious mutations. This finding is consistent with findings of previous studies demonstrating that arbovirus evolution is constrained to enable alternating infection of disparate vertebrate and arthropod hosts (*25*–*27*).

Additional work is needed to fully understand the roles that *R. aegyptiacus* bats and *O.* (*R.*) *faini* ticks play in maintaining KASV over time (*9*). Collection and separation of *O.* (*R.*) *faini* ticks according to life history stage, as well as experimental tick work, will be important in determining whether these ticks serve as amplification or reservoir hosts for KASV. Detection of infectious KASV in larval, nymphal, and adult stages of *O.* (*R.*) *faini* ticks would suggest that the virus is transtadially transmitted, and ticks serve as virus amplification hosts. Likewise, detection of KASV in nymphs and adults originating from KASV-artificially infected *O.* (*R.*) *faini* larvae would suggest the virus is transtadially transmitted from one generation to the next. Isolation of KASV in *O.* (*R.*) *faini* eggs found in nature or in larvae originating from KASV-artificially infected female ticks would indicate that the virus is transovarially transmitted and that ticks are reservoir hosts for the virus because a vertebrate host is not required for long-term virus survival. Similarly, a longitudinal ecologic investigation of KASV infection in *R. aegyptiacus* bats, as well as experimental KASV infection of captive bats, is critical in defining the relationship this virus has with its vertebrate host. Although KASV has previously been isolated from 2 wild-caught *R. aegyptiacus* bats (*6*), the isolation of actively replicating KASV over several days in experimentally infected *R. aegyptiacus* bats will confirm the ability of this bat species to serve as an amplification host for the virus. The detection of KASV in oral, rectal, or urogenital shedding collected from experimentally infected or wild-caught *R. aegyptiacus* bats will not only provide evidence that these bats are reservoir hosts of the virus but also indicate that they are capable of transmitting the virus to humans that encroach upon their habitat.

Although no human cases of KASV infection have been reported since the initial UVRI-associated cases described in 1977 (*6*), surveillance of populations at risk for KASV infection has never been conducted. Miners, herders, tourists, and researchers often frequent mines and caves occupied by large colonies of *R. aegyptiacus* bats. Entry into *R. aegyptiacu*s bat–inhabited environments has been linked to the spillover of several pathogenic agents into the human population, including Marburg virus (*11*,*28*,*29*), Sosuga virus (*30*,*31*), and *Borrelia* spirochetes (*13*). Similarly, humans who enter environments occupied by *R. aegyptiacus* bats and *O.* (*R.*) *faini* ticks are likely to be at risk for KASV infection. Surveillance of these at-risk populations for evidence of active or past infection KASV infection is needed to determine the true burden of KASV infection in humans.

Appendix Additional information on human-pathogenic Kasokero virus in field-collected ticks.
