# Supplementary material for: Human-Pathogenic Kasokero Virus in Field-Collected Ticks
Source: Emerg Infect Dis. 2020 Dec;26(12):2944–50. doi: 10.3201/eid2612.202411 (PMC7706932; doi:10.3201/eid2612.202411)
Supplement: Appendix — Additional information on human-pathogenic Kasokero virus in field-collected ticks. [file 20-2411-Techapp-s1.pdf]

Article DOI: https://doi.org/10.3201/eid2612.202411

Human-Pathogenic Kasokero Virus in Field-Collected Ticks

Appendix

Appendix Table 1. Quantitative reverse transcriptase PCR primers and probes

Assay	Oligo name	Sequence, 5′–3′
KASV N, 105-bp amplicon	Forward primer	GGACATTGACTCTCAGACTTC
	Reverse primer	GTCCAGGCACACTCATAAAT
	Probe	*56-FAM*-AGCAGTCAT-*ZEN*-CGCAGCCACCAGAAA-*3IABkFQ*
Shone tick 16S rRNA	Forward primer	CTGCTCAATGATTTTTTAAATTGCTGTGGT
	Reverse primer	CCGGTCTGAACTCAGATCAAGTAGGA
	Probe	*6FAM*-AAATAGTTTGCGACCTCGATGTTGGATTAGGAT-*BHQ1*
Applied Biosystems Eukaryotic 18S rRNA Endogenous Control Kit (VIC®/TAMRA probe, primer limited), Thermo Fisher Scientific	Not available	Not available

Appendix Table 2. Kasokero virus–specific tiling primers for genome amplification

Oligo name	Sequence, 5′–3′	Pool no.
KASV_S_1_LEFT	CAAAGACAGACGTGCCGCTTAC	1
KASV_S_1_RIGHT	AGAATCTGTTCAGCCTCAGGCA	1
KASV_S_3_LEFT	AGGAAGAAAAGACTTGGTGGTGT	1
KASV_S_3_RIGHT	GACTCTCCCTGTGCAACACTTG	1
KASV_S_5_LEFT	TCGTTTGCAAGCTCCAATTTTAACG	1
KASV_S_5_RIGHT	TATTTGGGGCAGCAACAACGTT	1
KASV_S_2_LEFT	GAACGATCCATGACCTGGTTCA	2
KASV_S_2_RIGHT	GCTCGATTTCGCTATCAAGCCA	2
KASV_S_4_LEFT	ATGAGTGAGGATGCTGCCAAGA	2
KASV_S_4_RIGHT	TGCAACTGGGAATGCTCCAAAG	2
KASV_S_6_LEFT	ATCCCTGTGCCTCTATGGTGAC	2
KASV_S_6_RIGHT	ATGTTGCCGCATACCCCAATTT	2
KASV_M_1_LEFT	AAAGAAAGACTTGCGGCTACCC	3
KASV_M_1_RIGHT	CCGTTGGCCATTTGGAGTACAG	3
KASV_M_3_LEFT	ACATGGAAATCGACAATCTGGCA	3
KASV_M_3_RIGHT	GGCAACCTTTTCAATTTGGACGT	3
KASV_M_5_LEFT	TGTCCACTTCGTGATTGAGAGAAA	3
KASV_M_5_RIGHT	TGACCCAACTCTATAGCAGCCA	3
KASV_M_7_LEFT	AGGTCCAGCTGAAACACTGTCT	3
KASV_M_7_RIGHT	AATTGACCATGTCGTTGCAGCT	3
KASV_M_9_LEFT	ATCAGAAGGACACCTAGGCTGG	3
KASV_M_9_RIGHT	GTATGTGCAGTGTTCCCCAAGG	3
KASV_M_11_LEFT	GCAGGTTCGAGTACATAACGGG	3
KASV_M_11_RIGHT	TGCAATTCTCTTTGGGAGCAGT	3
KASV_M_13_LEFT	ATGCATTAGTCTTTGATGACATTACAAGT	3
KASV_M_13_RIGHT	AAATTGCGAGTGAAACCTGGCA	3
KASV_M_15_LEFT	CTGTGTTGGCCTGGAACTTGAA	3
KASV_M_15_RIGHT	CAGAAAGGCTTTCTCCTCTGCC	3
KASV_M_2_LEFT	TGGGTGGAAATAAAACTGGAGTGA	4
KASV_M_2_RIGHT	GCAAACTTCTGTCCTTGAGGCA	4
KASV_M_4_LEFT	GCCACTACCAAAGGTACCACCA	4
KASV_M_4_RIGHT	CTTCTCTGCAAACCATGTGCCT	4
KASV_M_6_LEFT	ACAAGTCATTTCCAGATGCCAAAGT	4
KASV_M_6_RIGHT	AGTGAAGTCTGTGCTGTTTGCC	4
KASV_M_8_LEFT	GCATCACTCTGCAGGTCCTTTC	4
KASV_M_8_RIGHT	AGAGCAGGACTACAAACCACGT	4
KASV_M_10_LEFT	TGAACCGTCAAAAGAACCTCACA	4

Oligo name	Sequence, 5′–3′	Pool no.
KASV_M_10_RIGHT	ATCATCGCAGCCACAGTTCTTG	4
KASV_M_12_LEFT	AGTTTGTGAAGTCGTGGAAGCG	4
KASV_M_12_RIGHT	GTGCATGTTGTCCAGTCTCCAG	4
KASV_M_14_LEFT	AGAGCTGAATTCCAAAAAGGTTAACC	4
KASV_M_14_RIGHT	CTCCCCCTTCACAGGTCTGATT	4
KASV_M_16_LEFT	AGCTTCCTCAGACTGTTCAAGAGA	4
KASV_M_16_RIGHT	AGATATAGTGGCGGCTTACCCT	4
KASV_L_1_LEFT	CAAAGAAAGTAATCCCCCATATCCAGA	5
KASV_L_1_RIGHT	TTGCATTGTTCACCCAGACACC	5
KASV_L_3_LEFT	CACTTTCCAAGAGGAGCTGGAC	5
KASV_L_3_RIGHT	TTAGTTCACCGGTGACCACAAC	5
KASV_L_5_LEFT	AGCATAGACAAAAGACAGCTGCT	5
KASV_L_5_RIGHT	TCCGCAATATCCTTCCGTCTGA	5
KASV_L_7_LEFT	GGACACCGAGCAGCTTTCAATT	5
KASV_L_7_RIGHT	AAATCCTTGAGCGGCACATCAG	5
KASV_L_9_LEFT	TGCCGTGCAAGAAGTAAGTTCA	5
KASV_L_9_RIGHT	TCTCAGAAGGCGAGTTTTCAAGT	5
KASV_L_11_LEFT	TTGAAGTCTCGCTGGTAGAGGG	5
KASV_L_11_RIGHT	GGTTGGGCATCCTTCGTTCAAA	5
KASV_L_13_LEFT	CAAGCAATAAAACTGCCGACTGG	5
KASV_L_13_RIGHT	TGTCATTCTGCACTTTGAAGTTGGA	5
KASV_L_15_LEFT	ACTTTGAGTGGTACCAACAGCA	5
KASV_L_15_LEFT_NEW	ACTTTGAGTGGTACCAGCAGCA	5
KASV_L_15_RIGHT	ACCCCAAGCTTCTCTTCATGCT	5
KASV_L_15_RIGHT_NEW	ACCCCAAGCTTCTCTTCATGTT	5
KASV_L_17_LEFT	TGACGTACTCCTAGGTGATAGTATGC	5
KASV_L_17_LEFT_NEW	TGACGTACTACTAGGTGATAGTATAC	5
KASV_L_17_RIGHT	TCAGCAGTTTCTTCCAACACCT	5
KASV_L_17_RIGHT_NEW	TCAGCAGTTTCTTCTAACACCT	5
KASV_L_19_LEFT	AAAGGCAGCAAGGTCTTTCAGG	5
KASV_L_19_RIGHT	TCATGGACCTAGGTTCAGCAACA	5
KASV_L_21_LEFT	TCAAAGCTAACCTTCTACAACTGGAA	5
KASV_L_21_RIGHT	AGAGCCCTTCTTCCTCAATCTCT	5
KASV_L_23_LEFT	TGTCCCTGGTGTGTGCAGTATA	5
KASV_L_23_RIGHT	GAGGAAGCCTTTCACCATTGCT	5
KASV_L_25_LEFT	GTCACAGAGACTTGTTGGTCCA	5
KASV_L_25_RIGHT	TGCTGCTGGTATTTCCACTTGC	5
KASV_L_27_LEFT	TGCCACGTCTTCAGTTCTCACT	5
KASV_L_27_RIGHT	CATGCCTGCACGAGACTCAAAG	5
KASV_L_29_LEFT	CTCTATGTCCCCAAGTTTAGTGGTT	5
KASV_L_29_LEFT_NEW	CTCTATGTCCCCAAGTTCAGTGGCT	5
KASV_L_29_RIGHT	AATCTTTCTGATGCACGGGCTG	5
KASV_L_31_LEFT	GCTTCTTCCAGAAAAGCTCAGGA	5
KASV_L_31_RIGHT	TTTACCGTCAAGCACCTTTCCG	5
KASV_L_33_LEFT	TGAGTCAGAAACAAGAAGTCTGGC	5
KASV_L_33_RIGHT	ACACAAGACAGTCAAGTTATTCGCA	5
KASV_L_35_LEFT	TGCGTTTCAGCAATTTCTGTTATGC	5
KASV_L_35_RIGHT	TCCTGATGAGTCACCTGAGCTG	5
KASV_L_37_LEFT	AGCAAAGATGACCCAAAACTACTAAGT	5
KASV_L_37_RIGHT	GTGTGTGAGCCAGGAAGTGATG	5
KASV_L_39_LEFT	CGAAAGAGGAGTTGATCTGTGCC	5
KASV_L_39_RIGHT	TCTGACTACGGGCAATGACTGT	5
KASV_L_41_LEFT	GGATCGAACTAACCAAGGTGCG	5
KASV_L_41_RIGHT	CCGACAGGTTCCTTCACCTCTT	5
KASV_L_2_LEFT	ATACTGGGGAGGCTCGGTAGAA	6
KASV_L_2_RIGHT	AAGTGTCTCCTCCTCGTCCATG	6
KASV_L_4_LEFT	GCATTTGACATAAATTCACTTGGGCA	6
KASV_L_4_RIGHT	GCATAACGACCTCCTGCACAAG	6
KASV_L_6_LEFT	GTGTGTCTTTGGTTGTGAAATTGCA	6
KASV_L_6_RIGHT	ACGGCTTATAGAGTTCGGCACA	6
KASV_L_8_LEFT	CCATCCAAACGGTGTTTCCTGA	6
KASV_L_8_RIGHT	GCCTGCTTTCTTCTTGCCTGAT	6
KASV_L_10_LEFT	CGCACTGACTGGTGGATTGATG	6
KASV_L_10_RIGHT	TCCTTGATGATGCTACCGCTCT	6
KASV_L_12_LEFT	TTGAAAACTTCTCTGAAAAGCTGACAT	6
KASV_M_12_LEFT_NEW	AGT TTG TGA AGT TGT GGA GGC A	6
KASV_L_12_RIGHT	AGTGTCTTGGTGCCTCCTCTTT	6
KASV_L_14_LEFT	AACATCCATTATGACTTGGGATCAGA	6
KASV_L_14_RIGHT	TGAGCACCTTAATTCCTGCCCT	6

Oligo name	Sequence, 5′–3′	Pool no.
KASV_L_16_LEFT	TTGCAGTGCCTACAACACTACA	6
KASV_L_16_RIGHT	TGGACAGGACATTCTGCTCAGA	6
KASV_L_18_LEFT	TCAATAGCGACAGACAATTGATATTTGAT	6
KASV_L_18_RIGHT	GACCTCCAGCGTTTCAGTAAGG	6
KASV_L_20_LEFT	CCCTCCTGAATCAATCGAGAAAGC	6
KASV_L_20_RIGHT	TTGTGCCAATGACTTCAGCCAG	6
KASV_L_22_LEFT	AAAGACCTTGATGAGCGGGAGA	6
KASV_L_22_RIGHT	GCAGTTCTCTCAGTGAGGTCGA	6
KASV_L_22_RIGHT_NEW	GCA GTT CTC TTA GTG AAG TTG A	6
KASV_L_24_LEFT	AGGATTGGATGATTCAAGATCTGATTTCA	6
KASV_L_24_RIGHT	TGTGTGTTCGTCAAACCATCATCT	6
KASV_L_26_LEFT	AGTGGAATGATGCAGCAACTATTGA	6
KASV_L_26_RIGHT	CAGCATGTGTCACAGTCACCTG	6
KASV_L_28_LEFT	TCATGATGTCTCAGAGAATAACCCCT	6
KASV_L_28_RIGHT	TCAGGTAGATGGAGCATCAGCC	6
KASV_L_30_LEFT	AAGCCAGCAGTTTTCTAGTGGT	6
KASV_L_30_RIGHT	AGAGTGTCACTTGAGCAGGGTT	6
KASV_L_32_LEFT	ACCACTTTACACCATTTTTATGAAGTCTT	6
KASV_L_32_RIGHT	TAACGGCTTTGTGTGCATCCAG	6
KASV_L_34_LEFT	CGTTAGTGAACCTTATAAACGAGATAAACA	6
KASV_L_34_LEFT_NEW	CGTTAGTGAACCTTAATGAGATAAATAATG	6
KASV_L_34_RIGHT	AGGGCAATTACTGTCACACTCAG	6
KASV_L_34_RIGHT_NEW	AGG GCA ATC ACT GTC ACA CTC AG	6
KASV_L_36_LEFT	AAACCCTGTTTAGGCACAACCG	6
KASV_L_36_RIGHT	TGAACTCTTCAGCTGCCTTCCT	6
KASV_L_38_LEFT	AGTTTATGAAGACACAAGGATAGTGCT	6
KASV_L_38_RIGHT	TGAACCCGGTTTCATCCAGGTA	6
KASV_L_40_LEFT	TGCAGAAATGTAATCAGTAGGACTGG	6
KASV_L_40_RIGHT	TTCATAAGCTCCCTCCACAGCA	6
KASV_L_42_LEFT	GGAAAGAAAACATCAAGCTCTTGCA	6
KASV_L_42_RIGHT	ATACGAATGTTTGCCCTGCCAA	6

Appendix Table 3. Pairwise comparisons of *Kasokero orthonairovirus* nucleoprotein sequences*

Virus	1†	2	3	4	5	6	7	8	9	10	11	12	13	14
1		29.2	26.8	27.5	27.4	27.5	27.5	27.5	27.5	27.5	27.5	27.5	27.2	27.1
2	22.1		21.6	22.0	22.1	22.0	22.0	22.0	22.0	22.0	22.0	22.0	21.3	21.3
3	21.9	9.9		1.7	1.8	1.7	1.7	1.7	1.7	1.7	1.7	1.7	9.5	9.4
4	21.9	10.3	0.4		0.1	0.0	0.0	0.0	0.0	0.0	0.0	0.0	9.3	9.2
5	21.9	10.3	0.4	0.0		0.1	0.2	0.1	0.1	0.1	0.1	0.1	9.3	9.2
6	21.9	10.3	0.4	0.0	0.0		0.0	0.0	0.0	0.0	0.0	0.0	9.3	9.2
7	21.9	10.3	0.4	0.0	0.0	0.0		0.0	0.0	0.0	0.0	0.0	9.3	9.2
8	21.9	10.3	0.4	0.0	0.0	0.0	0.0		0.0	0.0	0.0	0.0	9.3	9.2
9	21.9	10.3	0.4	0.0	0.0	0.0	0.0	0.0		0.0	0.0	0.0	9.3	9.2
10	21.9	10.3	0.4	0.0	0.0	0.0	0.0	0.0	0.0		0.0	0.0	9.3	9.2
11	21.9	10.3	0.4	0.0	0.0	0.0	0.0	0.0	0.0	0.0		0.0	9.3	9.2
12	21.9	10.3	0.4	0.0	0.0	0.0	0.0	0.0	0.0	0.0	0.0		9.3	9.2
13	22.2	9.9	0.6	1.0	1.0	1.0	1.0	1.0	1.0	1.0	1.0	1.0		0.1
14	22.2	9.9	0.6	1.0	1.0	1.0	1.0	1.0	1.0	1.0	1.0	1.0	0.0	

*Upper diagonal: % nucleotide divergence; Lower diagonal: % amino acid divergence.
†1: Dak AnD 56 (Yogue virus), 2: 11SB17 (Leopards Hill virus), 3: Z-52963 (Kasokero virus; KASV), 4: UGA-Tick-20130490 (KASV), 5: UGA-Tick-20130501 (KASV), 6: UGA-Tick-20130502 (KASV), 7: UGA-Tick-20130503 (KASV), 8: UGA-Tick-20130508 (KASV), 9: UGA-Tick-20130514 (KASV), 10: UGA-Tick-20130524 (KASV), 11: UGA-Tick-20130525 (KASV), 12: UGA-Tick-20130533 (KASV), 13: UGA-Tick-20170048 (KASV), 14: UGA-Tick-20170128 (KASV).

Appendix Table 4. Pairwise comparisons of *Kasokero orthonairovirus* glycoprotein precursor sequences*

Virus	1†	2	3	8	12	13	14
1		38.4	38.7	38.5	38.5	38.4	38.4
2	39.6		34.3	34.3	34.3	34.3	34.4
3	39.5	31.9		2.2	2.2	2.1	2.1
8	39.5	31.8	0.9		0.0	0.6	0.6
12	39.5	31.8	0.9	0.0		0.6	0.6
13	39.5	31.8	0.8	0.3	0.3		0.2
14	39.5	31.8	0.8	0.3	0.3	0.0	

*Upper diagonal: % nucleotide divergence; Lower diagonal: % amino acid divergence.
†1: Dak AnD 56 (Yogue virus), 2: 11SB17 (Leopards Hill virus), 3: Z-52963 (Kasokero virus; KASV), 8: UGA-Tick-20130508 (KASV), 12: UGA-Tick-20130533 (KASV), 13: UGA-Tick-20170048 (KASV), 14: UGA-Tick-20170128 (KASV).

Appendix Table 5. Pairwise comparisons of *Kasokero orthonairovirus* RNA-dependent RNA-polymerase sequences*

Virus	1†	2	3	8	12	13	14
1		31.2	30.3	30.4	30.3	30.5	30.5
2	23.9		26.9	26.8	26.7	26.7	26.7
3	23.1	16.1		2.2	2.1	12.1	12.2
8	23.2	16.1	0.7		0.1	12.1	12.1
12	23.1	16.0	0.6	0.2		12.0	12.0
13	23.2	15.9	2.6	2.5	2.4		0.1
14	23.2	15.9	2.5	2.5	2.4	0.1	

*Upper diagonal: % nucleotide divergence; Lower diagonal: % amino acid divergence.

†1: Dak AnD 56 (Yogue virus), 2: 11SB17 (Leopards Hill virus), 3: Z-52963 (Kasokero virus; KASV), 8: UGA-Tick-20130508(KASV) , 12: UGA-Tick-20130533 (KASV), 13: UGA-Tick-20170048 (KASV), 14: UGA-Tick-20170128 (KASV).
